# Compromised Air Quality and Healthcare Safety from Smoking inside Hospitals in Shantou, China

**DOI:** 10.1038/s41598-019-44295-z

**Published:** 2019-05-28

**Authors:** Jun Zeng, Dangui Zhang, Yindu Liu, Duanlong Zhao, Yunxuan Ou, Jiezhuang Fang, Shimin Zheng, Jianbin Yin, Sicheng Chen, Yiling Qiu, Zhenbin Qiu, Siping Luo, Hui Zhou, Ying Lin, William Ba-Thein

**Affiliations:** 1 Clinical Research Unit, Shantou University Medical College, Shantou, Guangdong P.R. China; 2 Present Address: Division of Endocrinology, The People’s Hospital of China Three Gorges University, The First People’s Hospital of Yichang, Yichang, Hubei P.R. China; 3 Research Center of Translational Medicine, Second Affiliated Hospital of Shantou University Medical College, Shantou, Guangdong P.R. China; 4 Undergraduate Research Training Program (UGRTP), Shantou University Medical College, Shantou, Guangdong P.R. China; 5 Department of Microbiology and Immunology, Shantou University Medical College, Shantou, Guangdong P.R. China

**Keywords:** Health services, Health policy, Environmental impact

## Abstract

Achieving smoke-free healthcare facilities remains a great challenge in countries with a high smoking prevalence and weak regulation. Assessment of the impact of environmental tobacco smoke (ETS) and its constituent PM_2.5_ on the air quality in Chinese hospitals has not been reported. In this study, we conducted air quality surveys by measuring real-time PM_2.5_ concentrations with Dylos Air Quality Monitors in five tertiary hospitals in Shantou, China during summer (July-August 2016) and winter (November-February 2017). Twenty-eight-day surveys inside the hospitals showed median PM_2.5_ concentrations above the China Air Quality Standard in elevator lobbies (51.0 μg/m^3^, IQR 34.5–91.7), restrooms (40.2, 27.1–70.3), and corridors (36.5, 23.0–77.4). Evidence of tobacco smoking was significantly associated with PM_2.5_ spikes observed in all the survey locations, contributing to the air quality undesirable for health in 49.1% of total survey hours or 29.3% of summer and 75.4% of winter survey hours inside the buildings, and 33.5%, 25.7%, and 6.8% of survey hours in doctor offices, nurse stations, and patient rooms, respectively. In conclusion, smoking inside hospitals induces PM_2.5_ spikes that significantly compromise the air quality and impose significant health risk to the hospital inhabitants. Reinforcing comprehensive smoking ban with the vested interest of all stakeholders followed by creative disciplinary actions are suggested to ensure healthcare safety.

## Introduction

Tobacco smoking generates environmental tobacco smoke (ETS) that includes toxic substances and carcinogens in the form of gases and particulate matters including PM_2.5_ (particulate matter of ≤2.5 μm) and pollutes the immediate surroundings of the smoker^1^. Exposure to ETS is causally linked to numerous diseases in infants and children (such as asthma attacks, respiratory and ear infections, and sudden infant death syndrome, SIDS), pregnant women (such as infant mortality from low-birth weight), and adults (such as coronary heart disease, stroke, and lung cancer)^2^. Independently from the effects of ETS, short-term (hours, days) or long-term (months, years) exposure to PM_2.5_ as well is associated with serious cardiopulmonary diseases^3–5^. Thus, the compounding effect of ETS and PM_2.5_ exposure can reduce the life expectancy in the exposed population, especially the susceptible groups—the elderly, children, and those with preexisting lung or heart diseases^2^. Smoking in healthcare facilities is therefore legally banned in many countries^6^ and yet achieving truly smoke-free healthcare environment remains a great challenge for countries like China with high smoking prevalence and weak regulation^7,8^.

In the face of declining global smoking prevalence, China is still witnessing a high adult smoking prevalence at 27.7% (52.1% of men and 2.7% of women) and estimated 387 million smokers in a population of 1.4 billion^9^, with a high consumption rate (≥20 cigarettes per smoker per day)^8^. A nationwide study in China estimated that 673,000 premature deaths in 2005 were attributable to smoking^10^. Widespread smoking is still a prevailing problem in Chinese hospitals^7^ and among the hospital employees^11^ despite the nationwide smoking ban in healthcare facilities since 2011^12^. We also observed during our pilot surveys that visitors, patients, and hospital employees smoked inside the hospitals (unpublished observation). Although even transient pollution with ETS from smoking may impose health risk^4^ to non-smoking populations especially patients and healthcare workers, not only is smoking in hospitals poorly regulated but it is tolerated as well by many healthcare providers and administrators, who themselves are in fact smokers in China^7^.

A part of the reason why tobacco control efforts in hospitals in China and some other countries have so far been not so successful could be due to lack of public awareness about the gravity of passive exposure to ETS. This study aimed to investigate the impact of environmental tobacco smoke on the hospital air quality in five regional referral hospitals by monitoring real-time PM_2.5_, the widely-used proxy marker of ETS^13^.

## Methods

### Study design and study sites

We conducted air quality surveys by measuring real-time PM_2.5_ concentrations in five regional referral hospitals including three specialist hospitals (Hosp. A–C; 360 average beds) and two general hospitals (Hosp. D and E; 1,658 average beds) in Shantou, Guangdong, China during summer (July-August 2016) and winter (November-February 2017).

### Ethics statement

The study was approved by the ethics committee of Shantou University Medical College and the participating clinical departments in the hospitals. All methods were performed in accordance with the relevant guidelines and regulations.

### PM_2.5_ measuring device

Two Dylos Air Quality Monitors (DC1700pro, Dylos Corp. CA, U.S.A.) were used for real-time measurement of PM_2.5_ concentration in the air. Dylos is a laser photometry-based device, using a small fan to draw in air and particles and funnel them through a sensing zone, where particles are detected with a laser beam. The Dylos reports the number of particles in two sizes (>0.5 μm and >2.5 μm) every minute. The number of particles between 0.5–2.5 μm (i.e., PM_2.5_) was calculated by subtracting the 2.5 μm fraction from the total 0.5 μm count. The Dylos monitors are pre-calibrated and recalibrated annually by the manufacturer and this study was done within the valid calibration period.

### PM_2.5_ surveys

Real-time PM_2.5_ concentrations were measured by two air survey methods: “mobile air survey” for the public places outside and inside the hospital buildings, and “stationary air survey” for inside the clinical wards.

In mobile air survey, one Dylos in the continuous operation mode and one digital hygro-thermometer were placed in a net attached to a backpack, carried by a study staff who walked around the hospital premises and paused for at least 3 min (to achieve stable counts as one record of measurement by the Dylos) in each predesignated sampling point. The mobile air survey was done in an inconspicuous manner not to disturb people’s normal behaviors as much as possible. The surveys were usually conducted during office hours (8:00 am–6:00 pm) at least twice on the same day and up to 20 times for each sampling point over 28 days during two survey periods (summer and winter) in all the study hospitals.

In stationary air survey, a pair of Dylos (in the continuous operation mode) and digital hygro-thermometer was placed for 3–5 days in each sampling point to collect PM_2.5_ concentrations in five clinical wards of one agreeing hospital (hospital D) over 48 days during the winter survey period.

### PM_2.5_ sources

Evidence of tobacco smoking (viz. smokers, smoldering and extinguished cigarette butts in the ashcans and on the floors, and tobacco smell) was recorded at each sampling point during the PM_2.5_ surveys. To prevent reading PM_2.5_ generated from non-tobacco sources, we set the sample collection points ≥1 meter away from any doors, windows, or obvious sources of PM_2.5_ such as heaters, coolers, air conditioners, and frequent human movement and normalized the PM_2.5_ measurements for humidity reading as mentioned hereafter.

### Data analysis

Using the software Dylos Logger (Ver 2.0), the recorded data in the Dylos were downloaded and the accompanying data (time, temperature, humidity, evidence of tobacco smoking, and code of sampling points) were transferred to a Microsoft Excel spreadsheet. Conversion of the PM_2.5_ particle count concentration of 0.1 cubic ft. (measured by the Dylos) into the mass concentration of μg/m^3^ was done using the formula: PM_2.5_ (μg/m^3^) = no. of particles × 3531.5 × 5.89E-7 as reported previously^14^. Because PM_2.5_ concentration measured by the laser photometry method can be affected by humidity, the formula was modified for humidity adjustment with a correction factor (CF)^14^ as follows: PM_2.5_ (μg/m^3^) = no. of particles × 3531.5 × 5.89E-7 × humidity (%) × CF. The mean PM_2.5_ count from 3-min recording at each sample collection point in the mobile air survey or the PM_2.5_ count from 1-min recording in the stationary air survey was reported as one record in this study.

PM_2.5_ mass concentrations were converted to Air Quality Index (AQI) values using the United States Environmental Protection Agency’s online AQI converter [https://www.airnow.gov/index.cfm?action=airnow.calculator].

### Statistical analysis

SPSS 22.0 (IBM SPSS Statistics, USA) was used to analyze and report all the statistics in this study. Non-normally distributed PM_2.5_ mass concentrations were reported as median and interquartile range (IQR) using boxplots. Differences between median PM_2.5_ values were analyzed by the Mann-Whitney test. Association between PM_2.5_ spikes and evidence of tobacco smoking was analyzed by χ² test and multiple logistic regression analysis.

### Reference standards for air quality and health risk assessment

The guideline for 24-h mean concentration of PM_2.5_ by the China National Standard (CN-AQS, 35 μg/m^3^)^15^ was used as the reference standard for air quality. Levels of health concern/risk were assessed following the United States Environmental Protection Agency (US EPA)^16^.

## Results

### Mobile air survey outside and inside the hospital buildings (outside the clinical wards)

**Figure 1:**
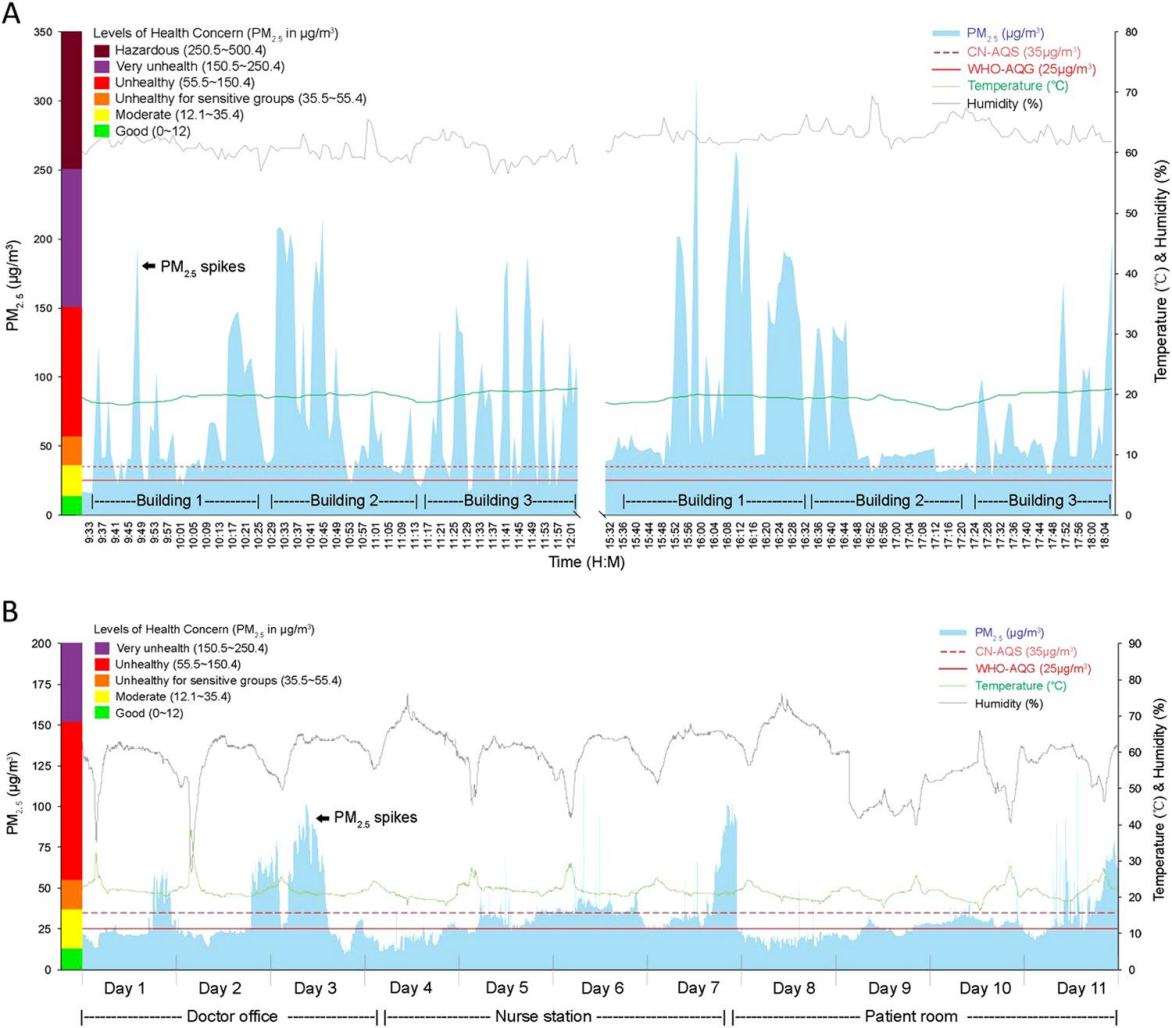
Representative graphs showing a mobile air survey of PM_2.5_ (**A**) and a stationary air survey of PM_2.5_ (**B**). (**A**) An air survey (two rounds per day in the same locations) is shown inside three hospital buildings (outside clinical wards) in hospital (**E**), with PM_2.5_ readings outside the hospital (environmental background reading) as gaps between the buildings. (**B**) An 11-day stationary air survey of PM_2.5_ in the doctor office, the nurse station, and a multi-patient room inside one clinical ward in hospital (**D**), where a PM_2.5_ monitor Dylos was placed for at least three days each in the same location for continuous measurements. Transient surges of PM_2.5_ concentration above the China Air Quality Standard (CN-AQS) are shown as PM_2.5_ spikes, with the WHO air quality guideline (WHO-AQG) as the international reference standard. The levels of health concern due to PM_2.5_ concentration was taken after the United States Environmental Protection Agency. See methods for the description of the mobile survey.

A representative one-day survey of PM_2.5_ is shown in Fig. 1A. A total of 4,061 records (from 394 sampling points, up to 338.4 h) were collected from all the hospitals as follows: outside the hospital buildings (226 records from 16 sampling points) and inside the hospital buildings (3,835 records, 378 sampling points) that included the main entrance hall (219 records, 36 sampling points), the stairways (2,000 records, 175 sampling points), the waiting rooms (150 records, 25 sampling points), the corridors (554 records, 44 sampling points), the restrooms (318 records, 20 sampling points), and the elevator lobbies (594 records, 78 sampling points).

**Figure 2:**
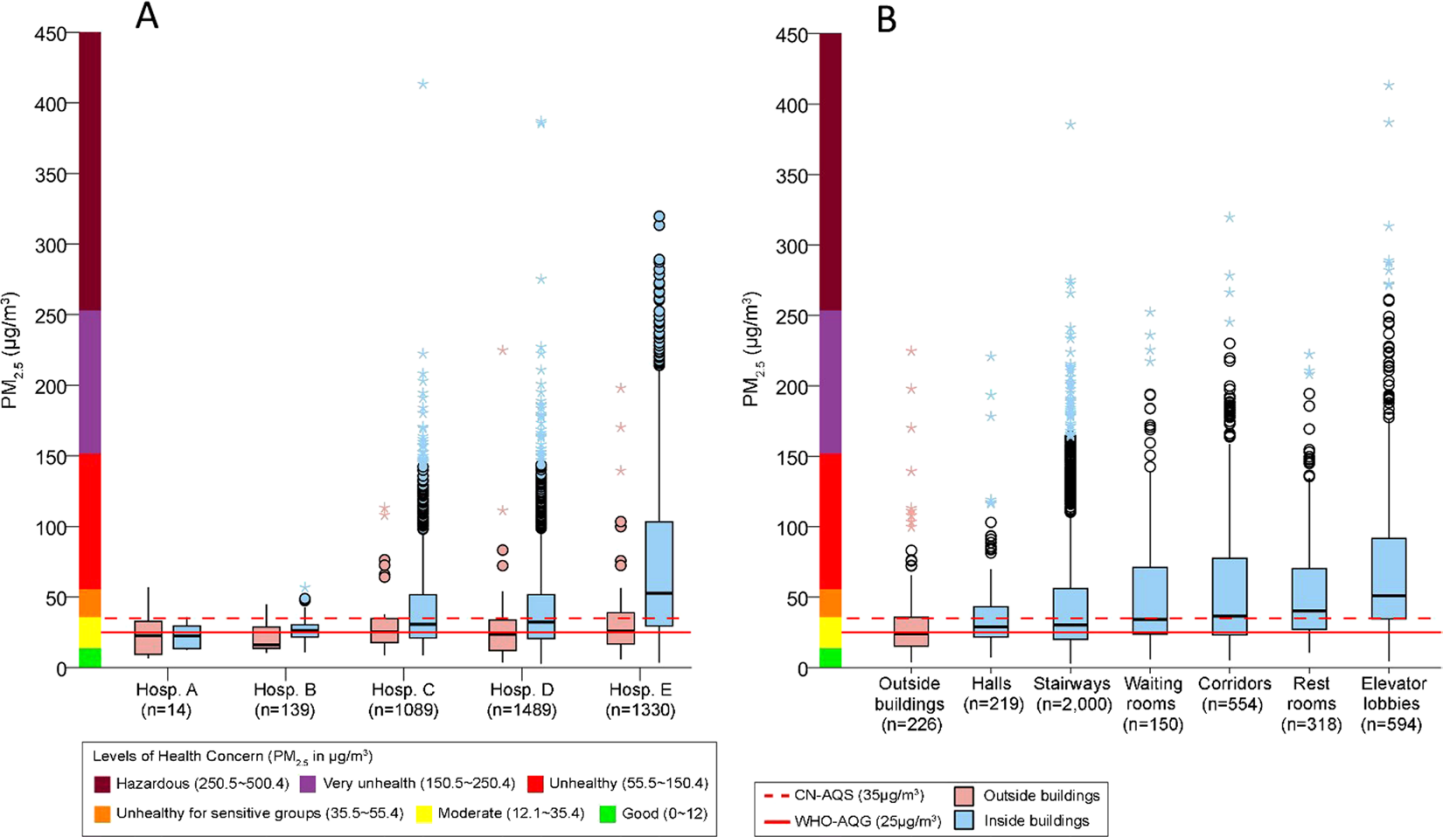
Mobile air surveys showing the overall PM_2.5_ concentrations (**A**) outside and inside the hospital buildings (outside the clinical wards) in five hospitals and (**B**) outside the hospital buildings in comparison with six different locations inside the hospital buildings (outside the clinical wards) of five hospitals, with the no. of records of PM_2.5_ measurement (n). Boxplot shows interquartile range, IQR (box) and median (horizontal bar in box) with outliers (1.5–3.0 × IQR above Q3, solid circles) and extreme values (>3.0 × IQR above Q3, stars). CN-AQS, the China air quality standard; WHO-AQG, the WHO air quality guideline.

The overall median PM_2.5_ concentration was significantly higher inside the hospital buildings than outside (34.8 μg/m^3^ [IQR 22.3–65.4] vs 24.0 μg/m^3^ [IQR 15.1–35.7]; *P* < 0.0001), especially in the general hospitals D and E (Fig. 2A). Mobile air surveys inside the hospital buildings in summer (2,190 records) and winter (1,645 records) showed a statistically significant difference in the median PM_2.5_ concentrations: 25.1 μg/m^3^ (IQR 18.2–40.2) in summer vs 54.6 μg/m^3^ (IQR 35.7–96.8) in winter, *P* < 0.0001.

Among the six defined locations inside the hospitals, three had the median PM_2.5_ concentrations above the CN-AQS (35 μg/m^3^), with the highest being the elevator lobbies (51.0 μg/m^3^, IQR 34.5–91.7), followed by the restrooms (40.2 μg/m^3^, IQR 27.1–70.3) and the corridors (36.5 μg/m^3^, IQR 23.0–77.4) (Fig. 2B). The presence of smokers, cigarette butts, smoldering cigarette butts, and tobacco smell was significantly more frequent in the stairways compared to the other sites (*P*s < 0.001, data not shown).

### Stationary air survey inside the clinical wards

**Figure 3:**
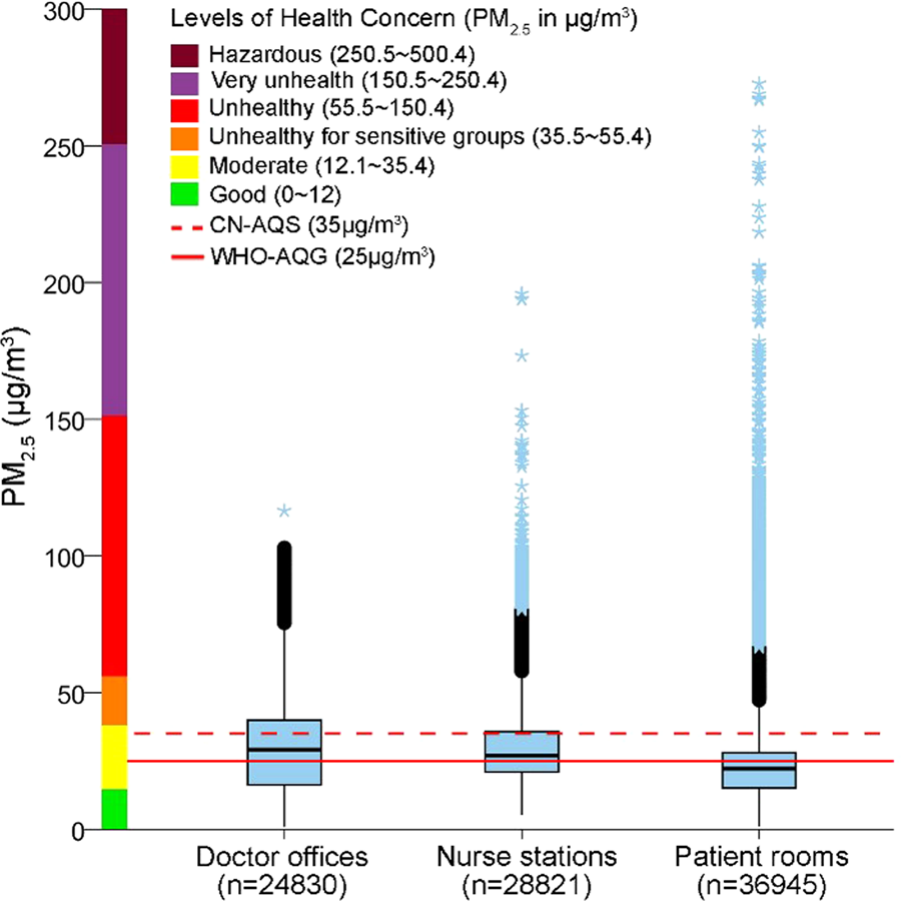
Stationary air surveys showing the overall median PM_2.5_ concentrations at three main locations inside the five clinical wards in hospital D and the no. of records of PM_2.5_ measurement (n). CN-AQS, the China air quality standard; WHO-AQG, the WHO air quality guideline.

A representative survey in one clinical ward over 11 days is shown in Fig. 1B. PM_2.5_ concentration was continuously monitored for at least three days in each sampling points in five clinical wards of the hospital D during the winter survey time. From a total of 90,596 records analyzed, the overall median PM_2.5_ concentration was 25.2 μg/m^3^ (IQR 17.1–32.9), with the highest concentration in the doctor offices (29.1 μg/m^3^, IQR 16.3–40.0), followed by the nurse stations (26.9 μg/m^3^, IQR 20.9–35.7), and the patient rooms (22.2 μg/m^3^, IQR 15.2–28.0) as shown in Fig. 3.

### Association between PM_2.5_ spikes and tobacco smoking

**Figure 4:**
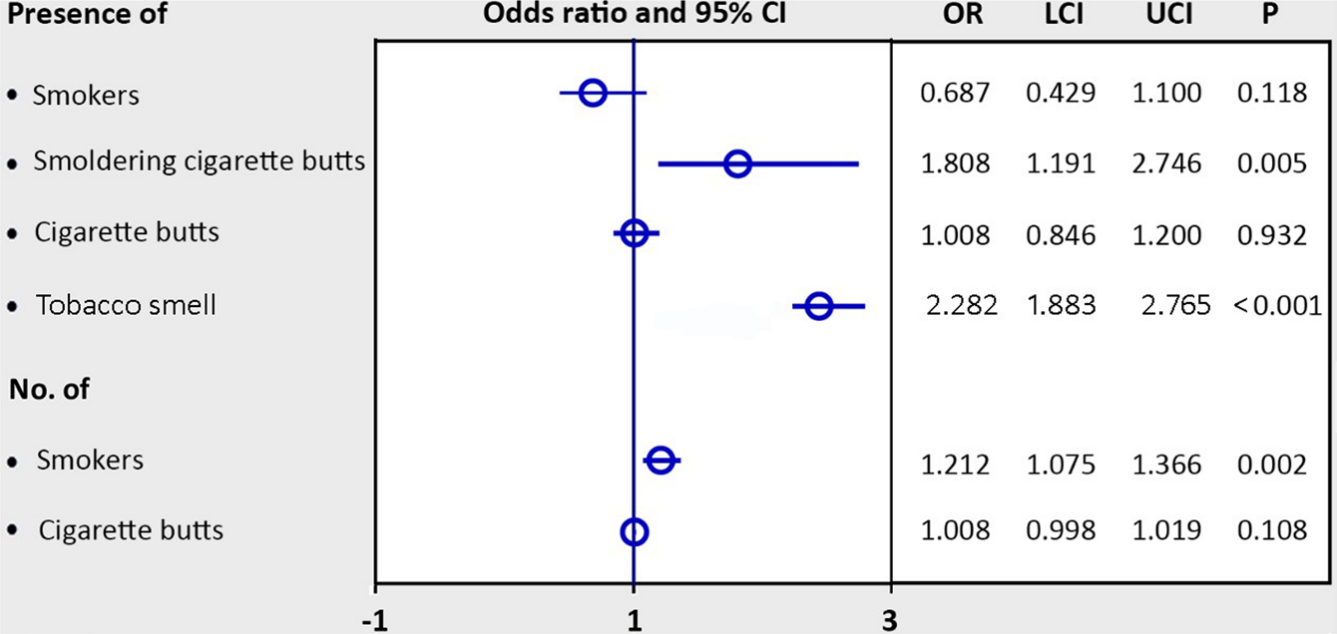
Evidence of tobacco smoking as predictors of PM_2.5_ spikes. Data were analyzed in multiple logistic regression models and presented as odds ratio (OR) with lower 95% confidence interval of OR (LCI) and upper 95% confidence interval of OR (UCI).

Evidence of tobacco smoking—the presence of smokers, extinguished or smoldering cigarette butts in the ashcans and on the floors, and tobacco smell—was present elsewhere on the property of all hospitals during our survey periods. PM_2.5_ spikes, which we defined herein as transient surges of particle count above the CN-AQS in the surveyed areas (Fig. 1A), were significantly associated with the evidence of smoking (*Ps* < 0.0001 by χ² test, Table 1). Multiple logistic regression analysis showed that the number of smokers and the presence of smoldering cigarette butts and tobacco smell were independently associated with higher odds (ORs 1.2, 1.8, and 2.3, respectively) of having PM_2.5_ spikes (Fig. 4).

**Table 1:** Association between PM_2.5_ spikes^#^ and evidence of smoking inside hospital buildings.

Presence of		PM_2.5_ spikes		*P* ^$^
		YES n = 1817 (%)	NO n = 1848 (%)	
Smokers	Yes (n = 1011)	674 (37.1)	337 (18.2)	<0.0001
	No (n = 2654)	1143 (62.9)	1511 (81.8)	
Smoldering cigarette butts	Yes (n = 1122)	749 (41.2)	373 (20.2)	<0.0001
	No (n = 2543)	1068 (58.8)	1475 (79.8)	
Cigarette butts (total)	Yes (n = 2450)	1316 (72.4)	1134 (61.4)	<0.0001
	No (n = 1215)	501 (27.6)	714 (38.6)	
Tobacco smell	Yes (n = 1772)	1109 (61.0)	663 (35.9)	<0.0001
	No (n = 1893)	708 (39.0)	1185 (64.1)	

^#^Transient surges of PM_2.5_ concentration above 35 μg/m^3^; ^$^ by χ² test.

Note: the data represented 3665 records of PM_2.5_ measurements and evidence of smoking inside hospital buildings in the mobile air survey.

### PM_2.5_ levels and health risk

As per the US EPA^16^, out of 319.6 h of mobile air survey over 28 days inside the hospital buildings, 49.1% of total survey hours or 29.3% of the summer and 75.4% of the winter survey hours could be classified as undesirable (PM_2.5_ > 35.4 μg/m^3^), comprising unhealthy for sensitive groups, unhealthy, very unhealthy, and hazardous levels of health concern.

**Figure 5:**
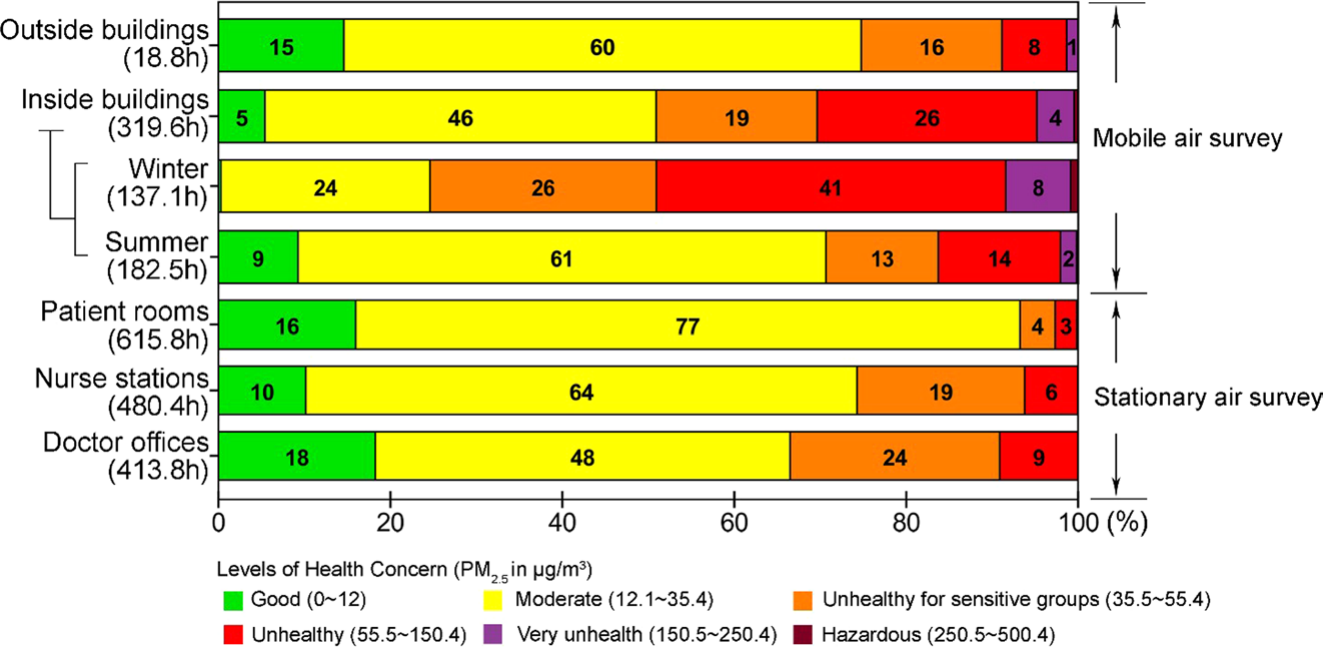
Levels of health concern based on the air quality inside five hospitals and inside five clinical wards in hospital D during the indicated survey times in hour (h). PM_2.5_ concentrations were converted to the levels of health concern using the Air Quality Index (AQI) Calculator from the United States Environmental Protection Agency.

The air surveys inside the clinical wards over 1,510 hours (in 48 days) also showed undesirable air quality especially in the doctor offices (33.5% of the survey hours), followed by the nurse stations (25.7%) and the patient rooms (6.8%) (Fig. 5).

## Discussion

Using PM_2.5_ as the ETS marker and the CN-AQS as the air quality standard, this study has demonstrated the poor air quality due to indoor smoking and associated health risks in patients, visitors, and hospital employees in the five regional referral hospitals in Shantou city, Guangdong province, China.

### Smoking as the source of PM_2.5_ spikes

The most significant observation in this study is the PM_2.5_ spikes (transient surges of particle count) due to ETS in both mobile and stationary air surveys. These spikes were up to 11.8 times above the CN-AQS (413 μg/m^3^), each spike lasting for up to 60 min especially when there was evidence of smoking and in the places with poor ventilation. A previous study has also shown that secondhand smoke remains in the air for a considerably long time (ca. 160 min) after indoor smoking^17^. Evidence of smoking without any other identifiable PM_2.5_ sources indicated that ETS was the exclusive source of PM_2.5_ spikes, contributing to the overall poor air quality observed in this study.

### Hospitals and wards with poor air quality

As per the CN-AQS, Shantou is one of the few cities that enjoy fairly-good air quality with the annual mean PM_2.5_ of 30 μg/m^3^ in 2016 and 29 μg/m^3^ in 2017 as reported by the Shantou government^18^ and supported by our finding of good air quality outside the hospitals over the survey period (i.e., 24 μg/m^3^ [IQR 15.1–35.7]). Nonetheless, significantly poor air quality was observed inside the hospitals and clinical wards. During our 28-day mobile air quality survey, 49.5% of total measurements were above the CN-AQS. The specialist hospitals (A–C) had a better air quality than the general hospitals (D and E) as they are generally smaller with better air conditioning or ventilation systems.

On the other hand, 20.7% of total measurements were above the CN-AQS in the stationary air survey inside the clinical wards. In fact, we noted considerably varied air quality in the clinical wards with some, e.g., the Burn Department in the hospital D, having a very good air quality (Supplementary Fig. S1), which suggests that for targeted interventions, hospital-wide investigation inside all the wards would be necessary.

### Polluted locations (hot spots)

Although all the survey locations inside the hospitals had higher PM_2.5_ concentrations than the outside, the elevator lobbies (the place most visitors frequented as a waiting area), the restrooms (a hideaway place for smokers), and the corridors where cigarette ashcans were laden with smoldering cigarette butts were three most polluted locations contributing to the undesirable level of health concern. The hospital stairways, the most favorite spot for smokers, had unexpectedly lower median PM_2.5_ concentrations due partially to good ventilation from the open structure of stairways but mostly to the smokers’ awareness of our repeat surveys and thus abstaining from smoking during the survey periods.

The clinical wards were also periodically polluted with PM_2.5_ spikes even at late night times, coinciding with the reported sighting of smokers (unpublished data from our survey with nurses and doctors). Although the highest median PM_2.5_ concentration was detected in the doctor offices, more frequent PM_2.5_ spikes were recorded in the nurse stations with a likely reason being their proximity to the ward entrance doors and the waiting places, the second most favorite spot for smokers. Despite low median PM_2.5_ concentrations, smoking in the patient rooms during unfettered hours could be noticeable from very tall PM_2.5_ spikes in the evening hours (Fig. 1b).

### Smoking populations in the hospitals

Even in the presence of no-smoking policy and no-smoking signs elsewhere, smoking was prevalent among the visitors, patients, and hospital employees. The smoking population of particular concern is the doctors on duty. Being an authoritative role model, they are sending the wrong message to the public and even countering no-smoking rules in the hospitals.

### Negative impact on healthcare safety

Healthcare safety primarily concerns with the safety of patients and healthcare workers. With known serious health risks from exposure to ETS and PM_2.5_, it is alarming that the air quality of nearly half of the total survey time or three-fourths of the winter time inside the hospital buildings was unhealthy to hazardous. The extent of poor air quality recorded in the clinical wards, although lower than that outside the wards, could, in fact, be more significant particularly for the patients with preexisting lung or heart problems because even brief exposure to ETS has immediate adverse effects on the cardiovascular system and can cause coronary heart disease and stroke^2^. On the other hand, healthcare workers who are being subject to intermittent but long-term ETS exposure could have an increase in their long-term risk of cardiopulmonary mortality^19^. Therefore, using PM_2.5_ values alone over any length of measurements, such as 24-h or annual mean, to assess health risk can be misleading where extreme PM_2.5_ spikes are present.

Taken all together, it is clear that tolerance to smoking inside hospitals and ignorance of health risks by the healthcare authorities could compromise not only healthcare but also the secondary objectives of hospital services—patient education and health promotion.

### Strengths and limitations of the study

The strength of this study lies on using portable PM_2.5_ monitor Dylos as both mobile and stationary air survey device and monitoring over extended periods, which enabled us to capture spatial, temporal, and seasonal dynamics of air quality on the hospital properties including all smoking hideouts. There are some limitations to consider in interpreting and generalizing our findings. Repeated air surveys in the same location somehow drew smokers’ attention, leading to avoidance of smoking in the survey areas, especially the clinical wards where the surveys were done with informed consent, therefore possibly under-estimating the true situation. The air quality data from five clinical wards of one hospital may not as well represent the situations in other clinical wards or hospitals. The assessment of health concern was based on PM_2.5_ data only; therefore, the risk associated with ETS could not be estimated from this study.

Another limitation is that lack of appropriate reference PM_2.5_ standards for real-time measurement led us to use the CN-AQS, which is based on 24-h mean concentration of PM_2.5_, as our reference. Therefore, it should be cautious in interpreting our health risk assessment that was based on the US EPA scale.

#### Suggestions

With lack of public awareness of health risk from passive smoking^20^, overcrowded hospitals, 14–64% of doctors being smokers^21^, and poor regulation in hospitals^22^, smoke-free hospitals in China could not be realized without the vested interest of all stakeholders. Redefining the comprehensive smoking ban on the hospital properties by replacing the usual no-smoking signs with customized signage to prevent sign-blindness, as suggested previously^23^, eliminating the designated smoking places on the hospital premises and grounds, continuing reinforcement and monitoring by hospital administrators and employees, and taking appropriate disciplinary actions on non-compliant smokers (especially the employees) could be a practically achievable approach. As an encouraging example, one clinical department with a responsible chief physician, who strictly adhered to the no-smoking policy and persistently reprimanded smokers, had an excellent air quality (data not shown).

Although our findings should be relevant to hospitals with similar situations in other parts of China or in any country, implementing any kind of intervention should be evidence-based and targeted at local smoking behaviors.

In conclusion, this study has demonstrated for the first time that poorly regulated indoor smoking in hospitals can generate PM_2.5_ spikes that compromise the hospital air quality and imposes significant health risk to healthcare workers and patients. Reinforcing comprehensive smoking ban with the vested interest of all stakeholders followed by creative disciplinary actions are suggested to ensure healthcare safety.

## Supplementary information

PM2.5 concentrations inside five clinical wards in the hospital D
