# Supplementary material for: Compromised Air Quality and Healthcare Safety from Smoking inside Hospitals in Shantou, China
Source: Sci Rep. 2019 May 28;9:7955. doi: 10.1038/s41598-019-44295-z (PMC6538634; doi:10.1038/s41598-019-44295-z)
Supplement: Supplementary file 1 — PM2.5 concentrations inside five clinical wards in the hospital D [file 41598_2019_44295_MOESM1_ESM.docx]

SUPPLEMENTAL MATERIAL

Manuscript title: Compromised Air Quality and Healthcare Safety from Smoking inside Hospitals in Shantou, China

Author list: Jun Zeng, Dangui Zhang, Yindu Liu, Duanlong Zhao, Yunxuan Ou, Jiezhuang Fang, Shimin Zheng, Jianbin Yin, Sicheng Chen, Yiling Qiu, Zhenbin Qiu, Siping Luo, Hui Zhou, Ying Lin, William Ba-Thein

**Figure S1. PM_2⋅5_ concentrations inside five clinical wards in the hospital D**


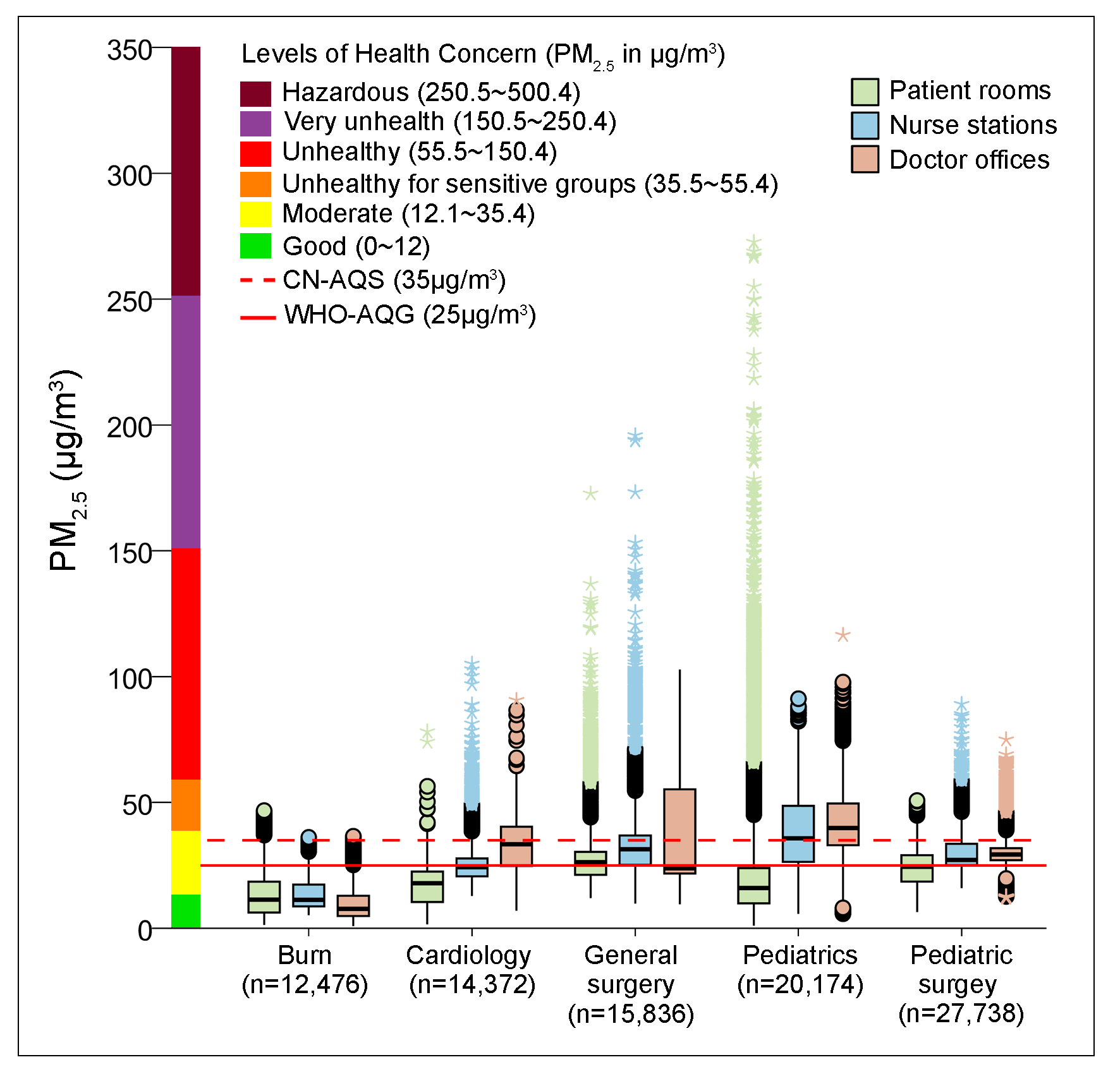


Stationary air surveys showing the overall median PM_2.5_ concentrations inside five clinical wards in hospital D and the no. of records of PM_2.5_ measurement (n). Boxplot shows interquartile range, IQR (box) and median (horizontal bar in box) with outliers (1.5–3.0 × IQR above Q3, solid circles) and extreme values (>3.0 × IQR above Q3, stars). CN-AQS, the China air quality standard; WHO-AQG, the WHO air quality guideline. PM_2.5_ concentrations were converted to the levels of health concern using the Air Quality Index (AQI) Calculator from the United States Environmental Protection Agency (US EPA).
